# ATTITUDES OF OLDER ADULTS TOWARD PHARMACEUTICAL DIRECT-TO-CONSUMER ADVERTISING

**DOI:** 10.1093/geroni/igz038.1717

**Published:** 2019-11-08

**Authors:** Ann M O’Hanlon, and Carlen L McLin

**Affiliations:** University of New Orleans, New Orleans, Louisiana, United States

## Abstract

According to national data, older adults view more television than any other age group. However, further research is needed about how older adults respond to programming. In particular, this study focused on the attitudes of older viewers toward pharmaceutical direct-to-consumer advertising (DTCA). Prior research has shown DTCA increases consumers’ demand for drugs, but less research has addressed how older consumers perceive such advertising. Researchers surveyed 293 older adults who attended Senior Centers throughout New Orleans about their television viewing and response to direct-to-consumer drug advertising. The sample was predominantly female (96%) and African American (87%) with a mean age of 76 years. The primary motivations selected for viewing television were to watch a favorite show, to be entertained, or learn information; older adults did not often endorse reasons of boredom, escapism, or relaxation. A majority of the sample (62%) perceived commercials as an interruption or annoying, and indicated they were uninfluenced by advertising (74%). When asked about direct-to-consumer advertising, 54 percent disapproved of such advertising, but 50% reported attending to such commercials when aired. Some older adults reported that advertisements on medicines could provide valuable information (46%), but also perceived the ads as confusing (57%) or alarming (61%). After viewing DTCA, older adults most often reported speaking to a doctor (46%) or seeking information (27%) about a drug, but some reported that they decreased (11%), increased (3%) or stopped (3%) taking medicine. The results provide additional information about how direct-to-consumer advertising is perceived by older adults and affects compliance behavior.

